# Clinical and Epidemiological Aspects of Acute Q Fever in Reunion Island over Fourteen Years: A Retrospective Cohort Study

**DOI:** 10.3390/microorganisms11102485

**Published:** 2023-10-03

**Authors:** Alexandra Aubin, Carole Eldin, Naël Zemali, Julien Jaubert, Yatrika Koumar, Marie-Pierre Moiton, Patrice Poubeau, Eric Braunberger, Patrick Gérardin, Antoine Bertolotti

**Affiliations:** 1 Service des Maladies Infectieuses—Dermatologie, Centre Hospitalier Universitaire (CHU) Réunion, BP 350, 97448 Saint Pierre, La Réunion, France; alexandra.aubin@chu-reunion.fr (A.A.); yatrika.koumar@chu-reunion.fr (Y.K.); antoine.bertolotti@chu-reunion.fr (A.B.); 2 Comité de Lutte Contre les Infections Nosocomiales (CLIN), Hôpital Nord, Chemin des Bourrély, 13015 Marseille, France; 3 Unité des Virus Emergents (UVE), Aix-Marseille Université, IRD 190 INSERM 1207 EFS-IRBA, 13005 Marseille, France; 4 Laboratoire de Microbiologie, CHU Réunion, BP 350, 97448 Saint Pierre, La Réunion, Francejulien-jaubert@chu-reunion.fr (J.J.); 5 Service des Maladies Infectieuses-Médecine Interne, CHU Réunion, 97400 Saint Denis, La Réunion, France; marie-pierre.moiton@chu-reunion.fr; 6 Service de Chirurgie Thoracique, CHU Réunion, 97400 Saint Denis, La Réunion, France; 7 Inserm CIC1410, CHU Réunion, BP 350, 97448 Saint Pierre, La Réunion, France; patrick.gerardin@chu-reunion.fr

**Keywords:** acute Q fever, *Coxiella burnetii*, pneumonia, Reunion Island, livestock

## Abstract

The clinical characteristics and epidemiology of Q fever in the Tropics are poorly described. We performed a retrospective cohort study of hospitalized cases between 2004 and 2017 in Reunion Island. Acute Q fever was defined in presence of a positive serology (phase II IgG ≥ 200 and phase II IgM ≥ 50), or a seroconversion (4-fold increase in phase II IgG between paired samples), or a positive PCR (blood or serum). Forty-two cases matched the diagnostic criteria. The most common clinical manifestations were fever (85.7%) and pulmonary symptoms (61.9%), including pneumonia (45.2%). Ninety percent of the patients were living in a farming area. Cumulative incidence was estimated at 9.3 per 100,000 inhabitants (95%CI: 6.4–12.1) with cases diagnosed yearly all throughout the study period except in 2006. Together with the seroprevalence figures, these data suggest that Q fever reaches low to moderate endemic levels on Reunion Island. As previously reported, pulmonary symptoms are in the foreground.

## 1. Introduction

*Coxiella burnetii* is an intracellular Gram-negative bacterium transmitted by inhalation of infected aerosols which causes acute or persistent infections. Small ruminants, like goats, are the main reservoir of *C. burnetii*. Clinical manifestations of acute Q fever are polymorphous and present most commonly as influenza-like illnesses (nasal congestion, runny nose, cough, body aches, and fatigue), pneumonia, or hepatitis. The primary infection, known as acute Q fever, is symptomatic in 30.0% of cases [1]. The main complications of the infection occur during “persistent” or “chronic Q fever” in patients with preexisting valvulopathy or vascular aneurysms. Chronic forms of the infection manifest as endocarditis of vascular infections and, more rarely, as osteoarticular diseases or chronic lymphadenitis [1]. Vascular infections are severe, with up to 18% mortality rates reported in the literature [1].

The epidemiology and clinical features of Q fever in the Tropics are poorly described except in Australia where the infection has been described initially. In French Guiana, a French Overseas territory, Q fever is the etiology of 38.5% of hospitalized community acquired pneumonia [2]. This very high prevalence is probably due to the multiple putative domestic and unusual wild animal reservoirs. The three-toed sloth is the first wild animal that has been identified to play a role in Q fever epidemiology in this territory [3,4]. More recently, the capybara, a wild rodent has also been identified as a potential reservoir [5]. The genotype circulating in French Guiana is unique, suggesting a specific virulence of this strain. 

Recently, a study in the South of Brazil found the *C. burnetii* seropositivity rate at 21.4% in patients consulting for suspicion of Dengue illness [6]. The disease has been reported in 11 states of this country, mostly Southeastern states, with an occupational character being reported in butchers, veterinarians, and farmers [7]. In Africa, most studies focus on the seroprevalence in ruminants and few data are available regarding clinical cases of acute Q fever. 

Reunion Island, a French overseas department of 2512 Km^2^ and 860,000 inhabitants, is located in the southwestern Indian Ocean region at the crossroad of migration routes connecting Europe, Madagascar, Africa, and Asia. The island is also a touristic destination, with 533,622 tourist arrivals in 2019 mainly practicing outdoor activities with potential exposure to zoonotic diseases. Other emerging zoonoses, like murine typhus have been previously reported on the island [8]. Some case reports have been published in the neighboring islands of the Comoros Archipelago and Mayotte, suggesting a significant circulation of the pathogen in this area [9,10]. 

The presence of *C. burnetii* in Reunion Island has been documented serologically for decades but was first reported in a scientific publication in 2014 among domestic ruminants [11]. The seroprevalence was 11.8% in cattle, 1.4% in sheep, and 13.4% in goats, and *C. burnetii* DNA was detected in vaginal swabs of cows (0.8%), sheep (4.4%), and goats (20%) [11]. In humans, the first clinical cases were diagnosed in 2007 within a couple of goat breeders after a massive epizootic in 2004 that decimated more than 2000 cattle. More recently, the human seroprevalence was estimated at 6.8% (95%CI 4.0–9.6%) with spatial correlates between seropositivity and presence of farms [12,13,14]. In addition, the seropositivity rate among parturient women was found as high as 20.1% (95%CI 17.7–22.5%) [13,14] and the seroprevalence of “probable infections” to be 4.7% (95%CI 3.4–5.9%), both definitions an association with adverse pregnancy outcomes for both [13,14]. However, no data about the clinical aspects of the disease in Reunion Island have been published so far. 

The aim of this retrospective cohort study was to describe the clinical features of acute Q fever diagnosed in Reunion Island hospitals over a fourteen-year period, to estimate its incidence, and to search for a geographic and seasonal pattern of acute Q fever cases. 

## 2. Materials and Methods

We performed a retrospective cohort analysis from the medical files of the four hospitals of Reunion Island (CHU Saint Pierre, CHU Saint Denis, CH Ouest Réunion, Groupe Hospitalier Est Reunion) between 2004 and 2017, using the search terms “Q fever” or “*Coxiella burnetii*”. This mere combination allowed for us to retrieve 145 files of presumed Q fever. We next sought diagnostic confirmation, applying the French consensual biological criteria for acute Q fever confirmation as follows: positive serology (defined as phase II IgG ≥ 200 and phase II IgM ≥ 50), seroconversion (4-fold increase in phase II IgG between paired samples), or a positive PCR for *Coxiella burnetii*.

**Figure 1:**
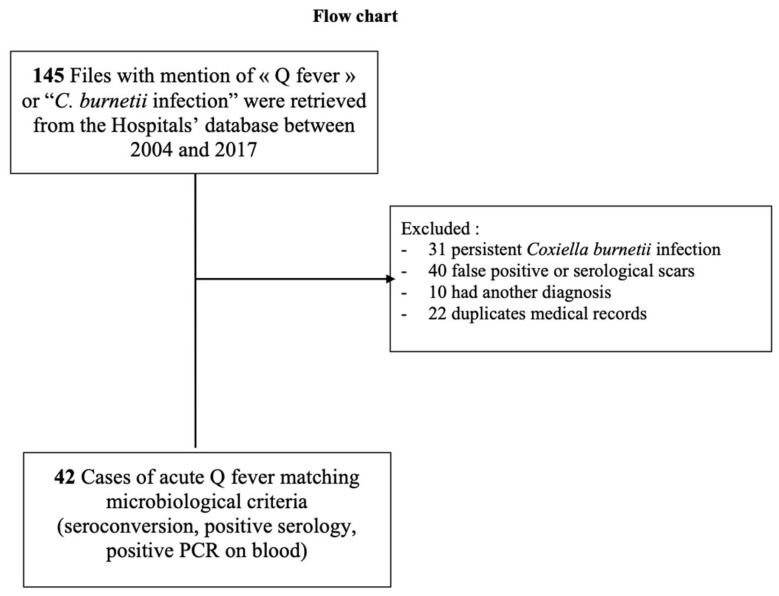
Study population for Q fever on Reunion Island, 2004–2017.

After the exclusion of 22 duplicates, 31 chronic Q fever, 40 false positive results, and 10 patients with differential diagnosis, we included 42 patients in the final analysis (Figure 1). Clinical and biological data were collected from medical files and completed, when possible by phone interviews of patients. Tests were performed on blood or serum either in the local laboratory of the hospital or in the National Reference Center for Q fever in Marseille, France.

Categorical variables were expressed as frequencies and percentages. The Chi-square test or Fisher’s exact test were used for comparison as appropriate. Continuous variables were expressed as medians with interquartile ranges. To reduce underreporting, which could be expected from a sampling bias due to the symptom-driven recruitment, the cumulative incidence of acute Q fever over the 2004–2017 period was estimated only for municipalities with reported cases. The cumulative incidence (i.e., the attack rate) was expressed as the sum of the observed cases divided by the local average population estimated by the French National Institute of Statistics (INSEE, Institut National de la Statistique et des Etudes Economiques) and compared with the local ruminant’s density [11].

Informed consent was obtained from the participants and data were treated anonymously. In accordance with French regulations, this observational retrospective study was registered within the National Institute of Health Data under the number MR 2411210519. No ethical approval was required due to the retrospective nature of data according to French legislation. The study was conducted according to the Declaration of Helsinki and the reference methodology MR-004 of the Commission Nationale Informatique et Libertés (CNIL).

## 3. Results

Overall, a total of 42 cases were classified as acute Q fever and analyzed. The median age was 47.5 years (37–59 years). Sixty-nine percent of patients were men, 17.0% reported spending time on a ruminant farm, and 26.2% being exposed to ruminants in their neighborhood. Smoking (40.5%) and diabetes mellitus (26.2%) were the most common medical histories (Table 1). Obesity was found in 19%. Regarding diagnosis, 29 patients had positive phase II IgG, 9 patients had positive phase II IgM, and 4 patients had seroconversion. 

Fever was the main clinical symptom (85.7%), followed by respiratory symptoms (61.9%) of which cough (42.9%), dyspnea (25.0%), and expectoration (23.8%). Headache was present in 35.7% of cases and 19% of patients had other neurological symptoms, 14.3% had unspecific cutaneous eruption. Abnormal chest images were found among 50.0% of the patients with interstitial opacities indicative of atypical pneumonia, while 45.2% of patients had a diagnosis of pneumonia in the medical files. Lung involvement was frequently associated with extra-pulmonary symptoms such as headache (35%) and digestive symptoms (26%). Sixty one percent of patients had fatigue and 38% reported arthromyalgia.

Biological investigations showed an inflammatory response pattern with elevated C-reactive protein levels (median 129.7 mg/L), leukocytosis in 64.3% of cases, and neutrophilia (69.4%). Positive anticardiolipin antibodies were detected in only one patient. The mean values of AST and ALT were low (35 and 38.5 UI/L, respectively) indicating no acute hepatitis. No renal impairment was observed. Almost ninety percent (88.6%) of the patients were treated, mainly with doxycycline (51.5%) or doxycycline and hydroxychloroquine in 15.1% of cases.

**Figure 2:**
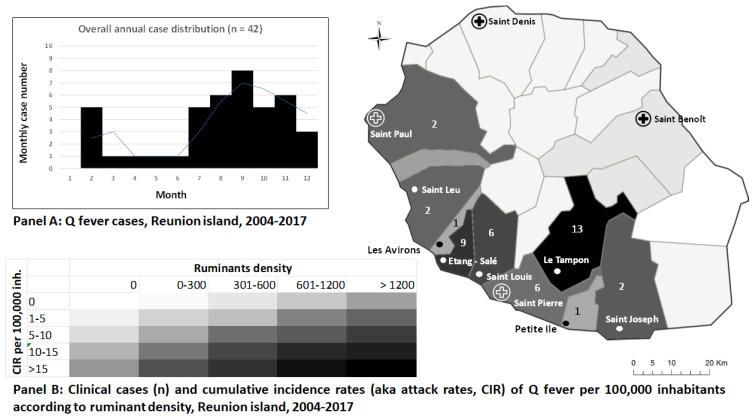
Temporo-spatial distribution of Q fever cases in Reunion Island, 2004–2017, with monthly cumulative cases numbers, (**Panel A**), heatmap of the spatial repartition of cases cumulative incidence rates and ruminants density (**Panel B**).

The cases were unrelated to each other and considered sporadic along the whole study period. They were reported each year except in 2006 and peaked in 2008 (n = 6), 2015 (n = 4), and 2016 (n = 5). A clear seasonal pattern was found, with most cases (45.2%, n = 19) occurring during the end of the austral winter (September to November), when trade winds are the strongest on Reunion Island, which represented a 2- to 4- month lag after lambing or calving (Figure 2, Panel A. When adding the two other whelping months at the start of winter, the period July to November represented 70% of cases (n = 30). Ninety percent of the patients (n = 38) were living in the South of Reunion Island, of whom 31.0% in Le Tampon (n = 13), 21% in Etang-Salé (n = 9), and 14% in Saint-Louis (n = 6) (Figure 2, Panel B.

The overall cumulative incidence was estimated at 9.3 per 100,000 inhabitants (95%CI: 6.4–12.1) but there were sharp discrepancies across municipalities; those exposed to ruminant farms and under the winds exhibiting the highest attack rates (Etang-Salé 61.9 per 100,000; Le Tampon 16.9 per 100,000; Saint Louis 11.1 per 100,000).

## 4. Discussion

The main clinical presentation of acute Q fever on Reunion Island is pneumonia, with nearly 62% of patients presenting with pulmonary symptoms and 50% having abnormal chest X ray of CT scan. This is consistent with the data obtained from French Guiana, Spain, and the Netherlands [15]. Conversely, in metropolitan France, hepatitis is more frequent, which probably reflects the genotypic differences in circulating strains, combined with diverse genetic and immunologic hosts factors [1]. Nineteen percent of patients had neurological symptoms, reminding us that the diagnosis of Q fever should be evoked in this setting, even if this presentation is rare. Headache is indeed a very frequent clinical sign during acute Q fever, with some patients reporting “the most severe pain they ever had” [1], which was reported here in 35.7% of cases. Cutaneous rash was also particularly frequent in our cohort (14%) compared to previous reports in the literature, with rates between 1% and 9% [1]. The male predominance of symptomatic cases (69%) that we found is well described in the literature, and hormonal and immunological factors have been hypothesized to explain this phenomenon [16]. It should be noted that almost 17% of patients had a valvular disease, which is a risk factor for Q fever endocarditis and suggests that patients should be followed up carefully after this episode of acute Q fever and that prophylactic treatment should be discussed. Regarding comorbidities, there is a high rate of obesity (19%), reflecting known higher rates in this territory than in metropolitan France (16% vs. 14%) and the same observation can be made about diabetes mellitus, for which the prevalence is twice the one of metropolitan France in Reunion Island.

This study has several limitations due to underreporting and hospital-based recruitment. The data collection was mainly retrospective, which may have caused an information bias, both in clinical and demographic data. Furthermore, antibodies titers were seldom retested, and very few blood specimens (n = 2) were sent to the French National Reference Center for confirmatory testing. In addition, cross reactions and co-infections especially with leptospirosis, may also interfere with Q fever serology, thus we excluded in this study patients with leptospirosis diagnosis (n = 2).

Many of the studies in tropical areas are serological surveys with no information on clinical data, so that it is the first study on acute Q fever clinical characteristics on Reunion Island. A weighted seroprevalence was estimated in 2009 at 6.8% in Réunion Island [12]. We estimated the incidence at 9.3 for 100,000 inhabitants confirming a possible *Coxiella burnetii* circulation on the island at low to moderate endemic rates. For example, the number of notifications in Europe was 0.2 cases per 100,000 inhabitants for 2017 with the highest notifications in Spain (0.8 cases per 100,000 inhabitants). The highest incidence described is in French Guiana with median value ranged from 26.3 in 2012 to 39.4 in 2011 per 100,000 inhabitants [15]. Our cumulative incidence is probably underestimated because of scarce notifications and asymptomatic or unspecific clinical symptoms that do not encourage serological testing and make the diagnosis of Q fever challenging. A prospective study should be considered with extended collection of data such as anticardiolipin antibodies and more systematical sending of samples to the French National Reference Center.

Interestingly, 90% of patients lived in the South of the island, in an area of livestock holdings (Figure 2, Panel B) [11], with a high proportion of them reporting being exposed to ruminants either directly (26.2%) or in their neighborhood (16.7%). Together with the absence of clustered transmission from a common source, this result suggests that classical reservoirs (livestock ruminants) are the main sources of human infection in this area. A previous study reported a *C. burnetii* seroprevalence of 16% in pregnant women hospitalized in the maternity of Le Tampon, the municipality which hosted 31% of our cases, suggesting that this area remains at risk for acquiring the infection [13]. 

Moreover, we observed a clear seasonal pattern of cases during austral winter, a season when trade winds are blowing from South-East. The same pattern of airborne transmission presumably from aerosolized birth products had been previously observed in ruminants, with an increase in *C. burnetii* infection when the farms were exposed to prevailing winds in the winter season and animals were not kept in their barn at night [11]. The airborne or wind-borne spread of *C. burnetii* from livestock holdings to the environment and humans has been documented for years and is recognized as the main route of *C. burnetii* transmission in endemic settings [17,18]. As a result, although in our retrospective observational study we cannot provide evidence beyond ecological correlates, we can only endorse and encourage the control measures and close monitoring of livestock by veterinarians aimed at mitigating the spread of *C. burnetii* infection on Reunion Island [19]. The isolation and genotyping of *C. burnetii* strains from Reunion Island would help the understanding of the epidemiology of the infection in this territory. Better awareness among clinicians about this disease in Reunion Island can help to prevent complications, improve public health interventions, and increase our knowledge about Q fever in Reunion Island.

## Figures and Tables

**Table 1:** Patient’s demographics, comorbidities, and clinical symptoms.

Variables	Total (n = 42)
Age, median (Q_1_–Q_3_), years	47.5 (37–59)
Age 18 to 45 years 46 to 82 years	18 (42.9%) 24 (57.1%)
Male gender	29 (69.0%)
Area-level exposure to ruminant farm ^†^	7 (16.7%)
Individual-level exposure to ruminants ^‡^	11 (26.2%)
Individual exposure to domestic animals ^#^	12 (28.6%)
History of smoking	17(40.5%)
Alcoholism	7 (16.7%)
Obesity	8 (19.0%)
Dyslipidemia	8 (19.0%)
High blood pressure	7 (16.7%)
Obliterative arterial disease of the lower limbs	5 (11.9%)
Stroke	3 (7.1%)
Valve disease	7 (16.7%)
Renal disease	2 (4.8%)
Diabetes mellitus	11 (26.2%)
Heart disease	5 (11.9%)
Immunodeficiency	4 (9.5%)
Bone infection	0
Fever	36 (85.7)
Median duration of fever (median (Q_1_–Q_3_), days	7 (5–15)
>14 days of fever	8 out 24 (33%)
Fatigue	26 (61.9%)
Arthromyalgia	16 (38.1%)
Pulmonary signs	26 (61.9%)
Dyspnea	7(25%)
Cough	18 (49.2%)
Sputum	10 (23.8%)
Abdominal symptoms	11 (26.2%)
Cutaneous rash	6 (14.3%)
Neurological symptoms	8 (19%)
Abnormal chest X ray or CT scan	21 (50%)
Median C-Reactive Protein (median (Q_1_–Q_3_), mg/L	129.7 (47.3–199.8)
Leukocytosis (Leukocytes > 10.0 × 10^9^/L)	27 (64.3%)
Leukopenia (Leukocytes < 3.5 × 10^9^/L)	3 (7%)
Thrombocytosis	2 (4.9%)
Thrombocytopenia	1 (2.4%)
Anemia	15 (35.7%)
Median (Q_1_–Q_3_) serum creatinin, μmol/L	81 (68–95)
Median ASAT, (Q_1_–Q_3_), IU/L	35 (23.5–57)
Median ALAT, (Q_1_–Q_3_), IU/L	38.5 (21.5–67.5)

^†^ Residence within 1 km radius from a ruminant farm. ^‡^ Occupational or incidental direct exposure to cattle, sheep, or goat herds due to occupation. ^#^ Direct exposure in daily life to pets, rodents, poultries, or goats dwelling at or around the house.

## Data Availability

Data are not available due to ethical restrictions.
